# Supplementary figures and images for: Effect of Roasting Level on the Development of Key Aroma-Active Compounds in Coffee
Source: Molecules. 2024 Oct 6;29(19):4723. doi: 10.3390/molecules29194723 (PMC11477549; doi:10.3390/molecules29194723)

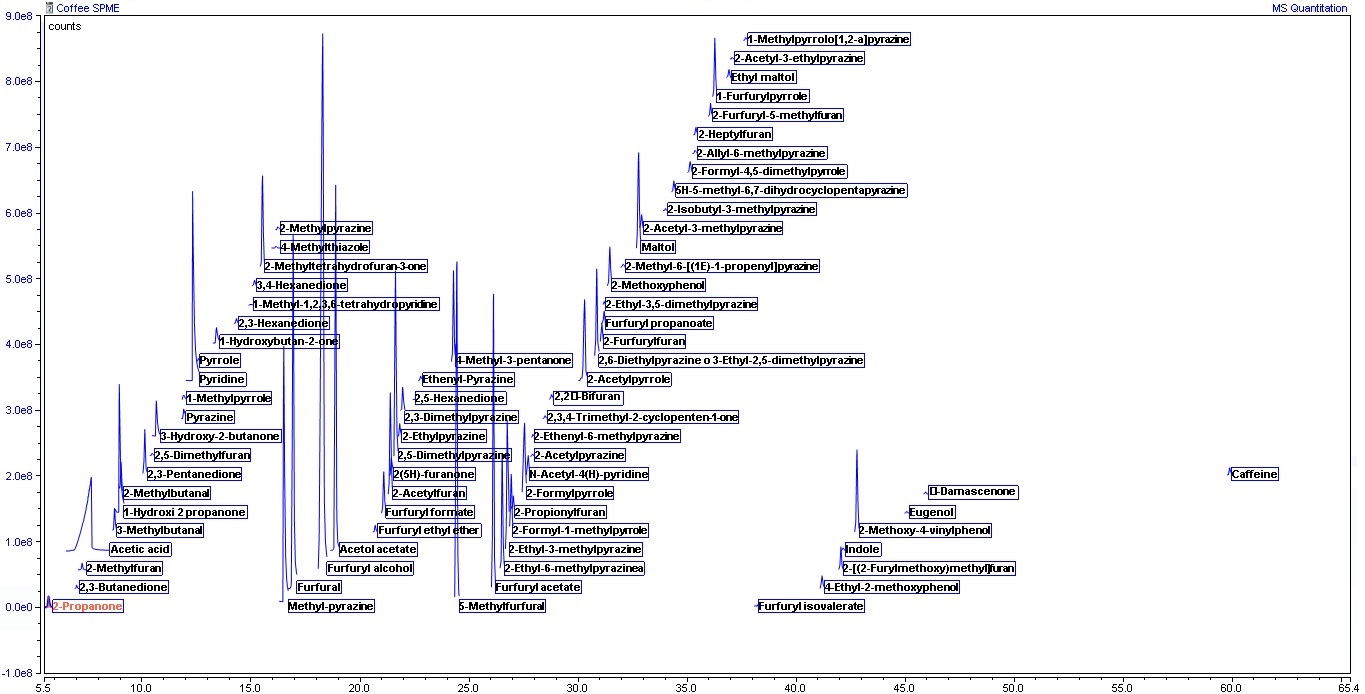

Supplement: Supplementary file 1 [file molecules-29-04723-s001.zip › Figure S1.jpg]
